# Supplementary figures and images for: Non-destructive insect metabarcoding for surveillance and biosecurity in citrus orchards: recording the good, the bad and the psyllids
Source: PeerJ. 2023 Aug 15;11:e15831. doi: 10.7717/peerj.15831 (PMC10437040; doi:10.7717/peerj.15831)

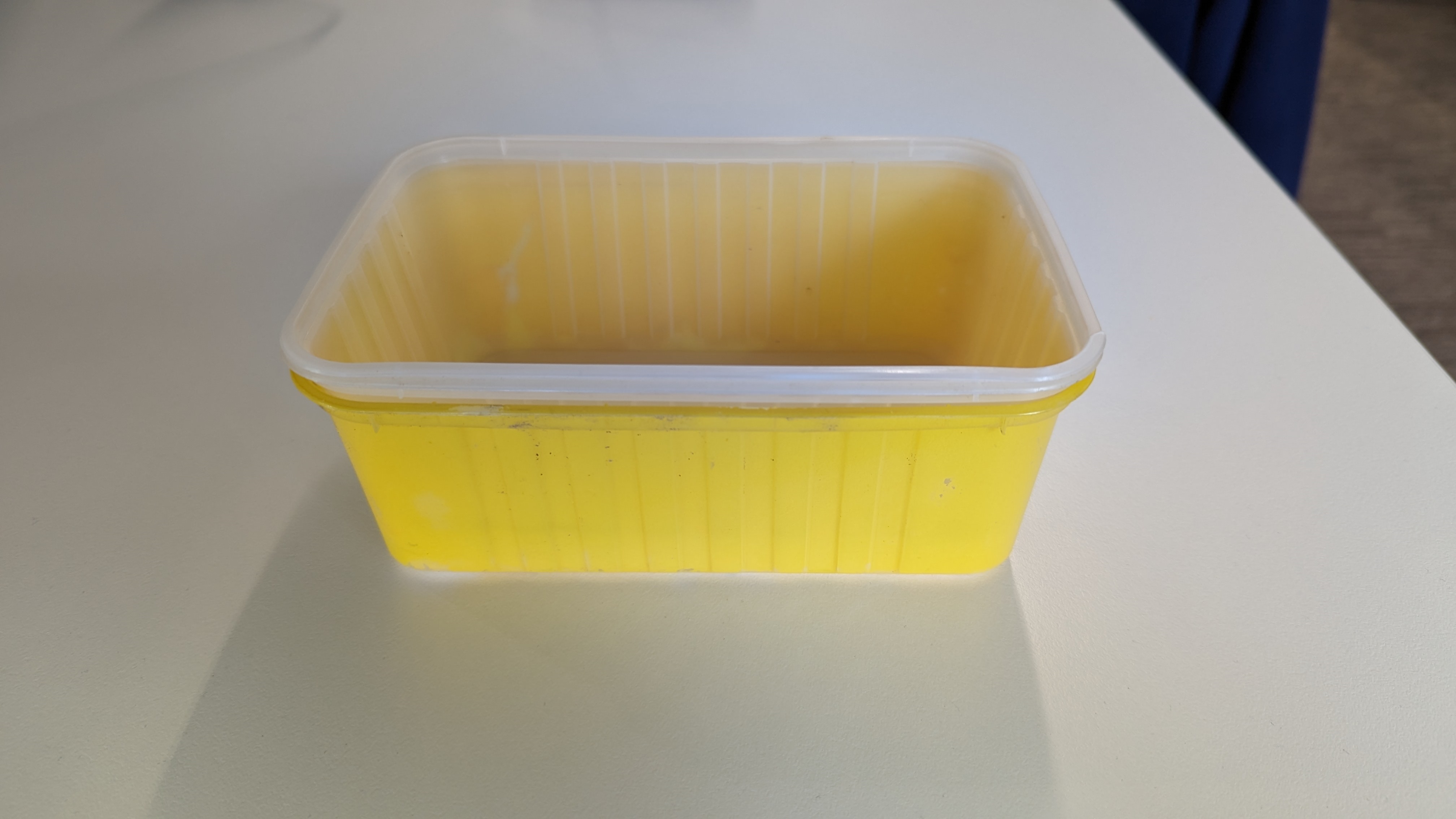

Supplement: Supplemental Information 2 — The white container was filled with a solution of Propylene Glycol and water (1:1), while the yellow container was secured to the ground using metal pegs. This enabled to collect the samples (from the white container) without moving the position of the trap, which remained secured to the ground (yellow container). [file peerj-11-15831-s002.jpg]

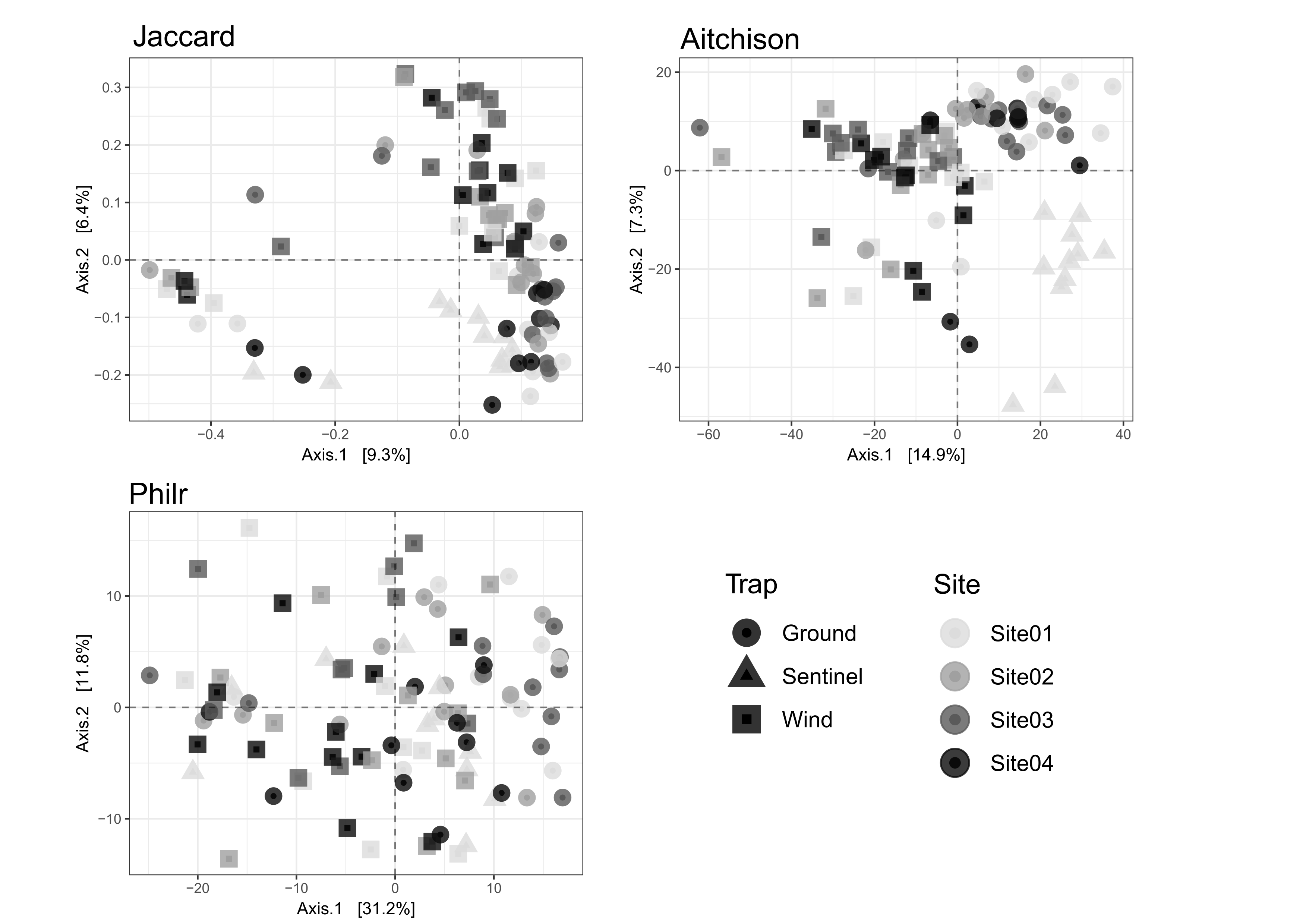

Supplement: Supplemental Information 3 — β-diversity PCoA plot (Jaccard, Aitchison, philr) comparing diversity between traps and sites. [file peerj-11-15831-s003.png]

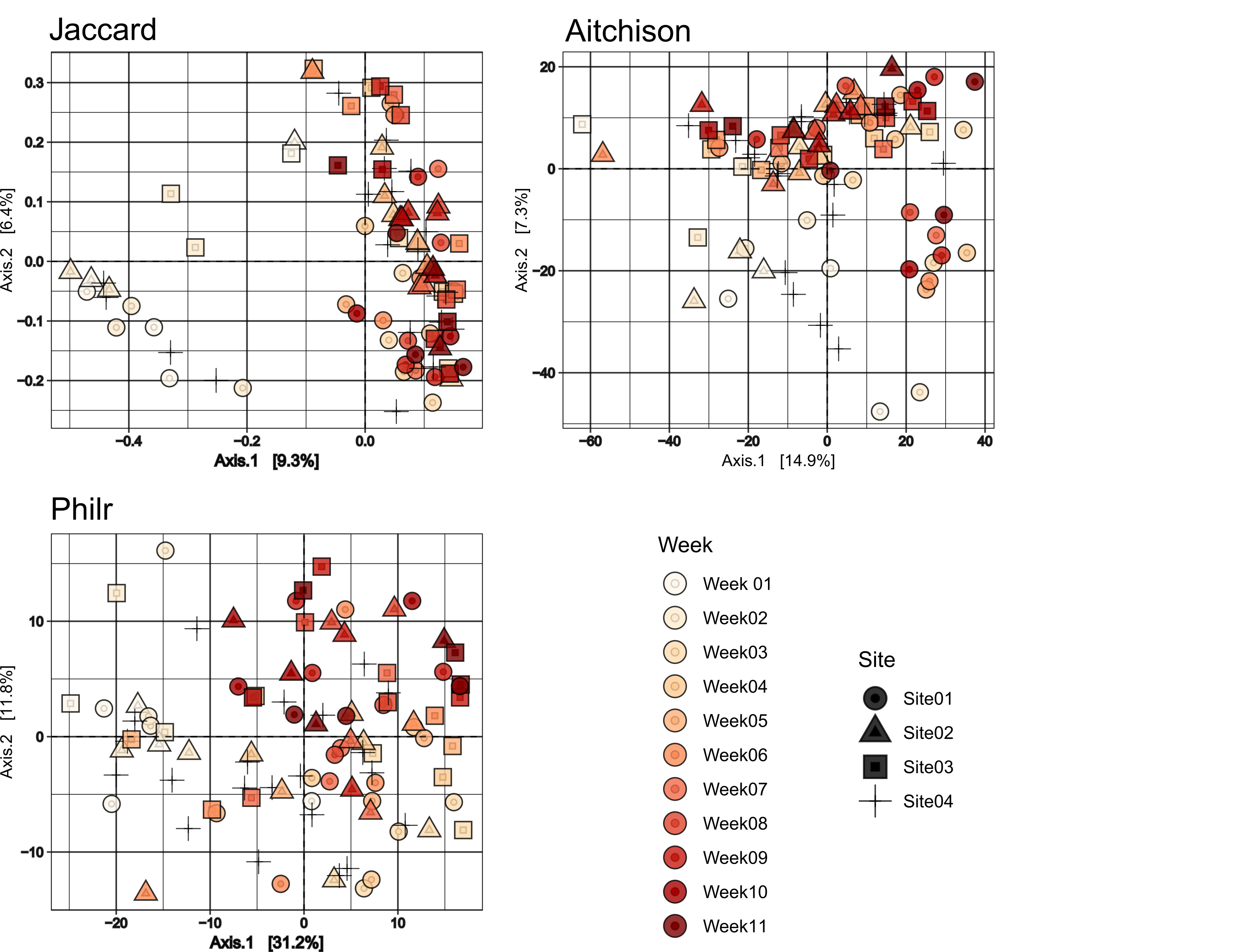

Supplement: Supplemental Information 4 — β-diversity PCoA plot (Jaccard, Aitchison, philr) comparing diversity between weeks and sites. [file peerj-11-15831-s004.png]

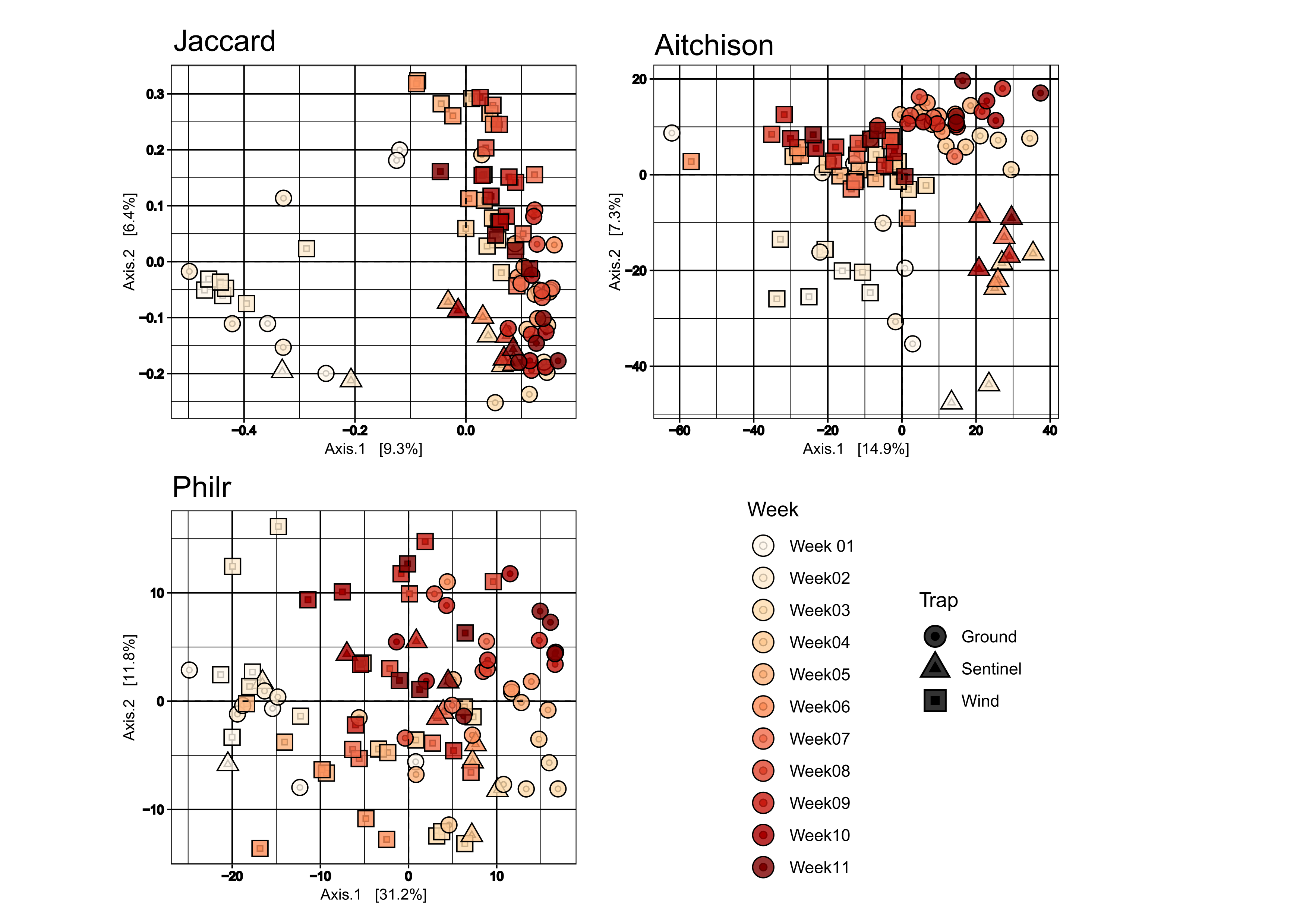

Supplement: Supplemental Information 5 — β-diversity PCoA plot (Jaccard, Aitchison, philr) comparing diversity between weeks and traps. [file peerj-11-15831-s005.png]
